# Breath Analysis for Lung Cancer Early Detection—A Clinical Study

**DOI:** 10.3390/metabo13121197

**Published:** 2023-12-12

**Authors:** Zhunan Jia, Velmurugan Thavasi, Thirumalai Venkatesan, Pyng Lee

**Affiliations:** 1 NUSNNI-Nanocore, National University of Singapore, Singapore 117411, Singapore; jzn596@gmail.com; 2 Center for Quantum Research and Technology, Homer L. Dodge Department of Physics and Astronomy, University of Oklahoma, Norman, OK 73019, USA; velmu@ou.edu; 3 Respiratory and Critical Care Medicine, National University Hospital, 1E Kent Ridge Road, Singapore 119228, Singapore

**Keywords:** breath test, volatile organic compound, point-of-care test, early diagnosis, cancer screening

## Abstract

This clinical study presents a comprehensive investigation into the utility of breath analysis as a non-invasive method for the early detection of lung cancer. The study enrolled 14 lung cancer patients, 14 non-lung cancer controls with diverse medical conditions, and 3 tuberculosis (TB) patients for biomarker discovery. Matching criteria including age, gender, smoking history, and comorbidities were strictly followed to ensure reliable comparisons. A systematic breath sampling protocol utilizing a BIO-VOC sampler was employed, followed by VOC analysis using Thermal Desorption–Gas Chromatography–Mass Spectrometry (TD-GC/MS). The resulting VOC profiles were subjected to stringent statistical analysis, including Orthogonal Projections to Latent Structures—Discriminant Analysis (OPLS-DA), Kruskal–Wallis test, and Receiver Operating Characteristic (ROC) analysis. Notably, 13 VOCs exhibited statistically significant differences between lung cancer patients and controls. The combination of eight VOCs (hexanal, heptanal, octanal, benzaldehyde, undecane, phenylacetaldehyde, decanal, and benzoic acid) demonstrated substantial discriminatory power with an area under the curve (AUC) of 0.85, a sensitivity of 82%, and a specificity of 76% in the discovery set. Validation in an independent cohort yielded an AUC of 0.78, a sensitivity of 78%, and a specificity of 64%. Further analysis revealed that elevated aldehyde levels in lung cancer patients’ breath could be attributed to overactivated Alcohol Dehydrogenase (ADH) pathways in cancerous tissues. Addressing methodological challenges, this study employed a matching of physiological and pathological confounders, controlled room air samples, and standardized breath sampling techniques. Despite the limitations, this study’s findings emphasize the potential of breath analysis as a diagnostic tool for lung cancer and suggest its utility in differentiating tuberculosis from lung cancer. However, further research and validation are warranted for the translation of these findings into clinical practice.

## 1. Introduction

Lung cancer stands as one of the most widespread and deadliest types of cancer globally, accounting for a substantial portion of cancer-related fatalities. It results from the uncontrolled proliferation of abnormal cells in the lung tissues, primarily triggered by exposure to carcinogens like tobacco smoke, air pollutants, and genetic predispositions. The early detection of lung cancer is paramount for effective treatment and enhanced patient prognosis. Conventional diagnostic techniques such as imaging methods and tissue biopsies have drawbacks in terms of invasiveness, expense, and accessibility. Consequently, there exists a critical need for non-invasive and precise diagnostic tools to identify lung cancer in its earliest stages [1].

Recent strides in medical research have led to the emergence of innovative diagnostic approaches, including breath tests, for the early detection of lung cancer. These tests scrutinize the volatile organic compounds (VOCs) found in exhaled breath, which can potentially serve as biomarkers indicative of the disease [2,3]. The breath test technique presents several advantages, including non-invasiveness, simplicity, and the capability to deliver real-time results. By pinpointing the specific patterns of VOCs associated with lung cancer, this novel breath test holds potential as a trustworthy and cost-effective screening tool that could significantly enhance early detection rates.

A range of existing methods has been employed in the quest for early lung cancer detection. Gas Chromatography–Mass Spectrometry (GC-MS) is a widely utilized technique known for its excellent sensitivity and specificity in identifying VOCs linked to lung cancer [4]. Nevertheless, GC-MS is time consuming and necessitates extensive sample preparation. Additionally, the sample container can introduce unpredictable effects on VOC concentration. The electric nose, or electronic nose, offers an alternative approach by utilizing sensor arrays to discern VOC patterns in exhaled breath [3]. While electric nose devices are relatively straightforward to use and provide real-time results, they may not possess the same level of precision and sensitivity as GC-MS. Liquid Chromatography–Mass Spectrometry (LC-MS) represents another valuable tool for identifying and quantifying VOCs. While LC-MS boasts exceptional resolution and sensitivity, its application in breath analysis is challenging due to the low levels of VOCs in breath samples. Furthermore, the HPPI-MS, an enhanced form of Single-Photon Ionization–Mass Spectrometry (SPI-MS), while showing promise in classifying healthy participants and cancer patients, is limited to detecting m/z and cannot identify specific VOCs [5]. Thus, despite their advantages, these existing methods may have limitations regarding sensitivity, specificity, and practicality for breath-based lung cancer detection.

In this study, we introduce a breath test that employs Thermal Desorption–Gas Chromatography–Mass Spectrometry (TD-GC/MS) and machine learning to detect cancer, particularly lung cancer. The paper outlines the design, methodology, sensitivity, accuracy, and potential clinical applications of the breath test system. Initial results demonstrate the feasibility and effectiveness of our breath test in identifying cancer patients, specifically those with lung cancer. This approach holds the potential to enhance lung cancer screening and contribute to improved patient outcomes through early detection and intervention.

## 2. Study Design

The study received ethical approval (DSRB Study Reference No: 2011/00380) from the Institutional Review Boards of the National University of Singapore, and all participants provided informed consent through signed documents. For the biomarker discovery phase (as detailed in Table 1), the research enlisted 14 individuals diagnosed with lung cancer, 14 non-lung cancer controls (comprising patients with conditions such as COPD, asthma, diabetes, and hypertension), and 3 patients with tuberculosis. It is worth noting that there were no statistically significant differences in terms of gender, age, and smoking history (*p* > 0.05) observed between the lung cancer group and the non-lung cancer controls. Furthermore, these two groups were closely matched in terms of comorbidities. In the subsequent validation set (as outlined in Table 2), the study included 18 participants diagnosed with lung cancer and 16 non-lung cancer controls. Importantly, this selection maintained a rigorous matching of gender, age, smoking history, and comorbidities between the two groups.

### 2.1. Breath Sampling Protocol

The breath samples were collected utilizing the BIO-VOC sampler, a procurement from Markes International United Kingdom, which is designed for this purpose. To ensure the utmost accuracy, the subject was instructed to exhale through a disposable mouthpiece until reaching the mid-tidal breath phase, displacing any residual environmental air within the sampling tube. This sampling tube is engineered to retain a fixed volume of breath and incorporates a one-way filter mechanism, preventing potential pathogenic contamination from the ambient air. The integrity of the sample was preserved by sealing the sampling tube with a PTFE plug. Following this initial collection, the sample was then transferred to a Tenax tube, sourced from SKC, United Kingdom, utilizing a precision plunger mechanism. This particular tube is airtight and houses specialized sorbent materials that are adept at trapping volatile organic compounds (VOCs) of interest. Subsequently, these collected Tenax tubes were safely stored at a controlled temperature of 4 °C until the analysis phase. Each Tenax tube was individually barcoded and assigned a unique identification for accurate subject delineation. For the lung cancer subjects, a total of three tubes of breath samples were collected per individual, ensuring a robust dataset for analysis. Conversely, control subjects provided two tubes of breath samples. Additionally, to account for any potential environmental contaminants, a sample of ambient air was also collected in parallel. This approach ensures that the subsequent analysis is not confounded by any extraneous factors, upholding the integrity and accuracy of the study’s findings.

### 2.2. GC/MS Details

Thermal Desorption–Gas Chromatography–Mass Spectrometry (TD-GC-MS) is a highly sensitive analytical technique used for the detection and identification of volatile and semi-volatile compounds in a wide range of samples. This method integrates three essential processes: Thermal Desorption, Gas Chromatography (GC), and Mass Spectrometry (MS).

First, thermal desorption involves heating the sample to release the volatile compounds from a solid or liquid matrix. This step allows for the efficient extraction of compounds that may be difficult to isolate through conventional extraction methods. The liberated compounds are then carried into the GC column by a flow of inert gas. In the GC phase, the individual components are separated based on their chemical properties, such as boiling point and polarity, as they interact with the stationary phase within the column. This separation process ensures that each compound is analyzed individually, reducing interference and enabling accurate identification. In the final step, the separated compounds are subjected to mass spectrometry, where they are ionized, fragmented, and then detected based on their mass-to-charge ratios. By comparing the obtained mass spectra with a database of known compounds, TD-GC-MS allows for the precise identification and quantification of the substances present in the sample. This technique finds widespread application in environmental analysis, food safety, forensics, and various other fields requiring sensitive and selective compound identification.

The analysis of volatile organic compounds (VOCs) was conducted through the utilization of Thermal Desorption–Gas Chromatography–Mass Spectrometry (TD-GC/MS). This sophisticated analytical technique employed state-of-the-art equipment, including a Unity Series 2 Thermal Desorber procured from Markes International Limited, in conjunction with a 6890 GC system from Agilent Technologies, interfaced with a 5973 MSD, also from Agilent Technologies. To initiate the process, the sampling tube underwent a pre-purge phase lasting 1 min, preparing it for optimal analysis. Subsequently, the collected breath gases underwent primary desorption at a temperature of 270 °C for a duration of 10 min, facilitated by a flow of helium gas. The total flow rate was set at 60 mL/min. The cold trap, a crucial component in the process, was maintained at a temperature of −10 °C. Following the primary desorption phase, the cold trap underwent a rapid heating process, ascending from −10 °C to 280 °C, and was maintained at this temperature for 5 min. The desorbed analytes were then introduced into an HP-5MS capillary column, measuring 60 m in length, 250 µm in diameter, and featuring a nominal thickness of 0.25 µm, all sourced from Agilent. This was facilitated through a transfer line, maintained at a temperature of 120 °C. The initial temperature of the GC oven was set at 40 °C. Upon sample injection, the oven temperature was progressively elevated, first to 140 °C at a rate of 5 °C/min, and then to 190 °C at an accelerated rate of 20 °C/min. It was further increased to 230 °C at a rate of 5 °C/min and held steady for a duration of 1 min. Finally, the temperature was raised to 300 °C at an expedited rate of 30 °C/min and maintained at this level for 5 min. The helium carrier gas flowed at a rate of 2 mL/min to facilitate the process. The detection of the analytes was achieved utilizing Mass Spectrometry in an electron impact mode, employing full scan monitoring across the range of 33–550 amu. The ion source temperature was set at 230 °C, while the quadrupole was maintained at a temperature of 150 °C. The transfer line temperature was calibrated to 280 °C, optimizing the entire process. The gain factor was consistently set at 1.58, ensuring precise and reliable detection throughout the analysis.

### 2.3. Statistical Analysis

Orthogonal Partial Least Squares Discriminant Analysis (OPLS-DA) is a statistical technique used in multivariate data analysis, particularly in the field of chemometrics and metabolomics. It is an extension of Partial Least Squares Discriminant Analysis (PLS-DA) that aims to improve the interpretability of the results. OPLS-DA is specifically designed to find the variation in the data that is correlated with the class labels (e.g., different groups or categories) while also separating unrelated variation, which is referred to as orthogonal variation. This makes it particularly useful in situations where there is complex, correlated, and noisy data, such as in biological studies where there might be many variables measured simultaneously.

The key principle of OPLS-DA lies in the separation of systematic variation into two components: one that is correlated with the class labels (predictive component) and the other that is orthogonal to the class labels (orthogonal component). This separation allows for a clearer interpretation of the variation in the data. The predictive component identifies the directions in the data space that are most relevant for discriminating between different classes, making it easier to understand which variables are driving the classification. Meanwhile, the orthogonal component captures variation that is unrelated to the class labels, effectively removing noise and confounding factors. This orthogonalization process enhances the interpretability of the model.

OPLS-DA is particularly valuable in cases where the number of variables (features) is much larger than the number of samples, a situation commonly encountered in high-dimensional datasets. By extracting the predictive and orthogonal components, OPLS-DA helps in identifying the important variables for the classification and understanding of the underlying structure in the data. This makes it a powerful tool for tasks like biomarker discovery, quality control, and other applications where distinguishing between different groups is crucial. However, it is important to note that, like any statistical technique, the results of OPLS-DA should be interpreted with care, taking into consideration the assumptions and potential limitations of the method.

**Figure 1:**
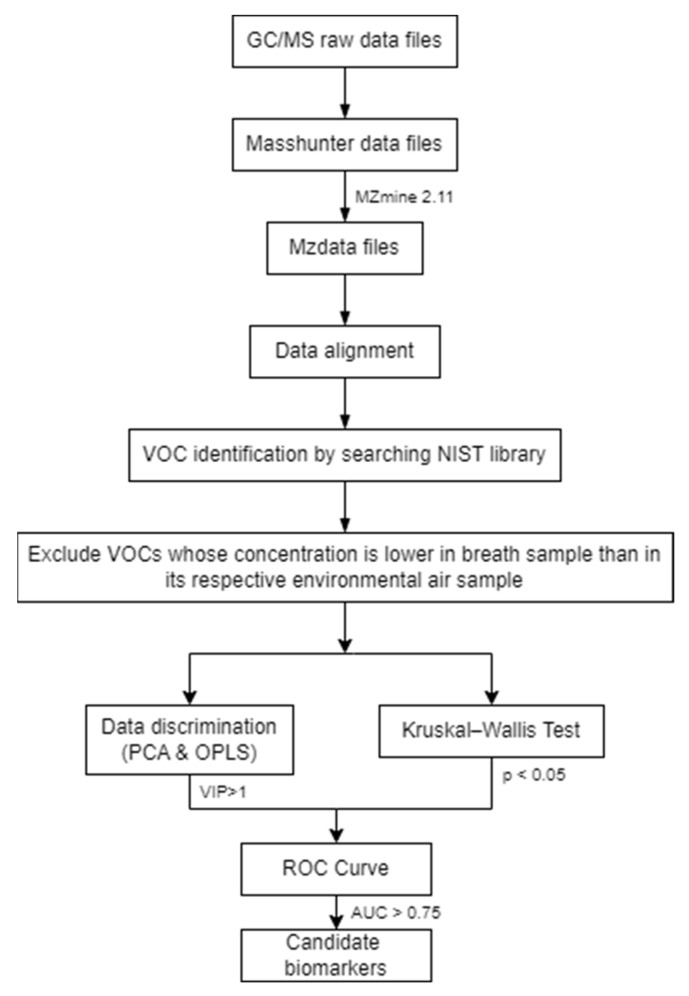
GC/MS data analysis workflow.

The handling of data files followed a systematic process. Initially, all data files were migrated into Masshunter data using the GC MSD Translator software provided by Agilent Technologies. Subsequently, they were exported into mzdata files, a format compatible with MZmine 2.11, enabling seamless peak alignment and normalization. For the identification of volatile organic compounds (VOCs), the NIST library was consulted, with only compounds exhibiting a match and reverse match score exceeding 700 being reported. Furthermore, to ensure robust analysis, the concentrations of VOCs in the samples were compared with those in the environmental samples. Compounds with concentrations lower than the environmental background were omitted from consideration. The next phase involved employing OPLS-DA, executed using SIMCA-P 13.0 by Umetrics. Additionally, Kruskal–Wallis tests and receiver operating characteristic (ROC) analyses were conducted using SPSS software (2016, Version 24.0) by IBM. The comprehensive workflow for biomarker screening is visually depicted in Figure 1.

Throughout the data acquisition process, three breath samples were collected from each case, whereas two samples were obtained from each control subject. During the subsequent data analysis, it was noted that the variance in samples from a single subject displayed variability among individuals. Detailed discussions regarding potential reasons for this phenomenon can be found in the dedicated discussion section. In the OPLS-DA analysis, rather than averaging multiple samples from one subject, each individual breath sample was treated as a distinct data point.

## 3. Results and Discussion

**Figure 2:**
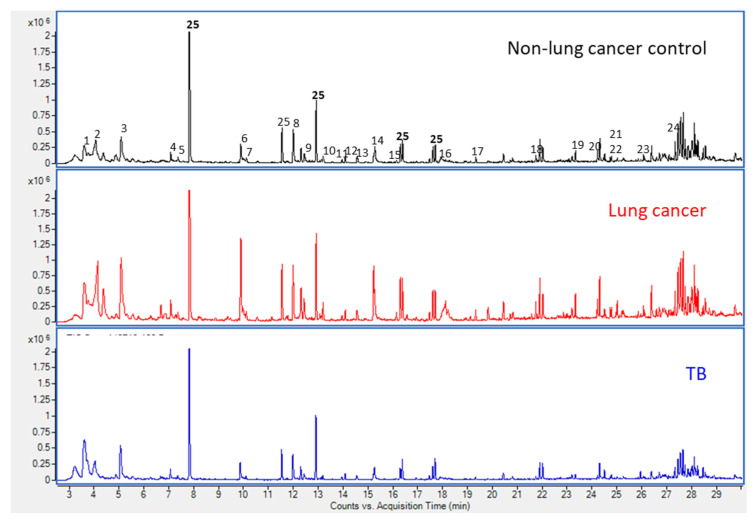
TIC of representative samples. (1: Acetone; 2: Acetic acid; 3: Dimethylsilanediol; 4: 4-Ethylbenzamide; 5: Hexanal; 6: Oxime-, methoxy-phenyl; 7: Heptanal; 8: Benzaldehyde; 9: Phenol; 10: Octanal; 11: Ethylhexanol; 12: D-Limonene; 13: Benzeneacetaldehyde; 14: Acetophenone; 15: Undecane; 16: Benzoic acid; 17: Decanal; 18: Undecanal; 19: Dodecanal; 20: 1-Dodecanol; 21: 1,2–15,16-Diepoxyhexadecane; 22: 2,4-Di-tert-butylphenol; 23: cis-9-Hexadecenal; 24: p-tert-Octylphenol Hexadecenal; 25: Compounds from GC/MS column).

**Figure 3:**
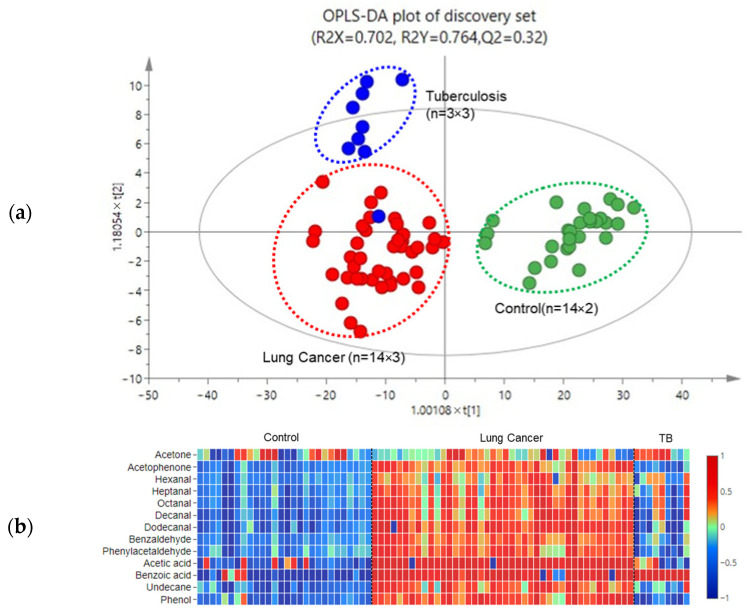
(**a**) OPLS-DA score plots of discovery set; (**b**) a color-coded map of 13 statistically differential compounds in the discovery set.

**Figure 4:**
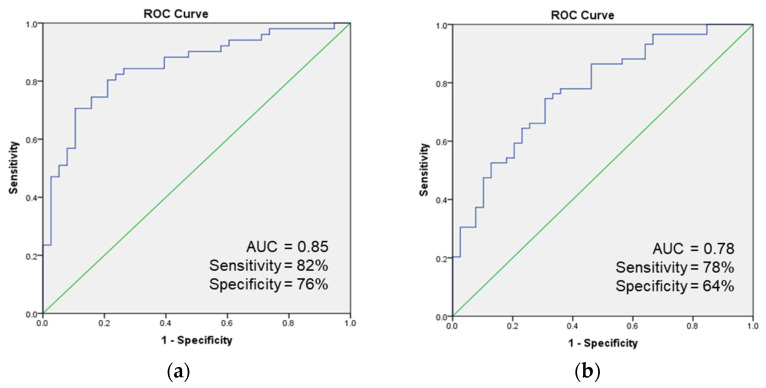
ROC curve of combined 8 biomarkers in (**a**) discovery set and (**b**) validation set.

Figure 2 shows the Total Ion Chromatograms (TIC) of a representative sample from each group, each peak is labeled with the compound name. Using the Kruskal–Wallis test and VIP calculation in the OPLS-DA model, a total of 13 compounds were found to be statistically different. Figure 3a is the OPLS-DA score plot of the VOCs from the discovery set. In this plot, each breath sample is represented by one dot. It shows a clear separation between lung cancer and control subjects, as well as between lung cancer and TB patients. The relative concentration of each VOC was linearly normalized into a range of −1 to 1 and plotted in a color-coded map shown in Figure 3b. A receiver operating characteristics (ROC) analysis was performed using the 13 compounds in the discovery set samples. Eight compounds with an AUC value above 0.75 (hexanal, heptanal, octanal, benzaldehyde, undecane, phenylacetaldehyde, decanal, and benzoic acid) were selected as a combined biomarker, and an ROC curve was plotted using them, shown in Figure 4a. The combined eight biomarkers achieved an AUC value of 0.85, a sensitivity of 82%, and a specificity of 76%. The validation set data were analyzed in the same way. Table 3 lists the fold change (lung cancer/control) and *p* value of the eight VOCs in both the discovery and validation sets. In the validation set, the combined eight biomarkers achieved an AUC of 0.78, a sensitivity of 78%, and a specificity of 64% (Figure 4b).

Seven aldehydes (including hexanal, heptanal, octanal, decanal, dodecanal, benzaldehyde, and phenylacetaldehyde) were found in elevated levels in lung cancer patients’ breath compared to those in non-lung cancer controls. This is possibly due to the increased levels of Alcohol Dehydrogenase (ADH) in cancer tissues. It is known that ADH is overactivated in cancer cells [6,7], which means a cancer cell has a greater ability to oxidize alcohol into aldehydes. Alcohols are products of alkanes by cytochrome p450; alkanes in turn are products of lipid peroxidation, a process which is also upregulated in cancer cells [8,9]. Similar to our results, hexanal heptanal and octanal were previously reported to be higher in lung cancer patients’ blood and breath samples [10,11,12]. Other aldehydes such as propanal, butanal, and nonanal were also found to be higher in lung cancer patients’ breath [13,14,15]. It is highly likely that these aldehydes are products of cancerous pathological processes. Benzealdehyde was found to be increased in lung cancer cell cultures [16,17]. An elevated level of one alkane, undecane in this study, is also in agreement with the literature [18,19,20,21]. Undecane is a by-product of lipid peroxidation by reactive oxygen species in cancer tissues. Breath acetic acid vapor was used as a biomarker for gastro-esophageal reflux disease [22] and cystic fibrosis [23]; it has not been reported to be associated with lung cancer before. Benzoic acid was found to be higher in both lung cancer patients’ breath and TB patients’ breath; the origin of benzoic acid is unknown. An increased level of benzoic acid, but not other VOCs, could be used to differentiate TB and lung cancer; however, this conclusion is limited by the small TB sample size in this study and needs further validation.

Despite the fact that many studies have produced lists of volatile biomarkers for lung cancer, none of these has been translated into clinics so far. The reason is the inconsistencies in the sampling method and study design [24]. Bikov et al. summarized the established methodological issues in breath research [25], and the European Respiratory Society has published a technical standard on breath sampling recently [26].

In the current study, we controlled several physiological and pathological confounders including age, gender, smoking history, and comorbidities. Isoprene, alkanes, and methylated alkanes were found to be related to age [16,17,18]. In many earlier studies, disease groups are significantly older than the control group [13,27,28,29,30]; this might have biased the results. Gender also has an effect on the breath VOC profile [31,32]. Smoking-related VOCs such as acetonitrile and benzene will also affect the results [32]. As one of the biggest risk factors for lung cancer, smoking history between case and control group should be closely matched to avoid any bias. Diseases other than the targeted one will also change the breath VOC profile; therefore, it is critical that control and case have matched comorbidities in order to find the VOCs specifically associated with the targeted disease.

During breath sampling, we collected the alveolar phase by using a BIO-VOC sampler. For diagnostic purposes, end-tidal breath is desired because it is more representative of the endogenous metabolism compared to airway dead space air, which is often affected by diet and bacteria. Some VOCs (such as carbonic acid, dimethyl ester, and methyl formate) were found to be significantly higher in end-tidal breath while methylene chloride and pentane, 3-ethy were lower in end-tidal breath compared to whole breath [33]. The use of sorbent traps minimizes sample loss and contamination during transportation and storage. Another important confounding factor is the environmental background. Room air—especially in clinics—may contain high levels of VOCs, and they vary from day to day. The degree of influence depends on the type of VOC, time, concentration of exposure, etc. Many studies have adopted inspiratory filters to reduce the effect of background VOCs [19,30,34,35]. However, it is not known yet how long it takes for the human body to equilibrate with filtered clean air. In the current study, we did not use an inspiratory filter due to limited resources. Instead, we controlled this confounder by taking a room air sample for each subject at the same time and location. VOCs with lower concentration in the breath than in the room air were excluded from further analysis.

The current study, while providing valuable insights, is not without its limitations, which should be taken into consideration. Firstly, we were unable to regulate the diet and exercise routines of the participants. These factors have the potential to influence the results, and their uncontrolled variability could introduce some degree of uncertainty. Secondly, we opted to gather three breath samples from each patient and two from each control subject. It is important to acknowledge that the reproducibility of breath samples can vary among individuals. This analysis showed that the analytical methods employed are consistent and reliable, showing no significant technical or systemic variations. However, it is worth noting that for certain other subjects, the reproducibility among different breath samples was less satisfactory. This discrepancy may be attributed to inconsistent breathing maneuvers among participants. This variability in breath sample reproducibility highlights the importance of considering individualized factors that may impact the reliability of breath-based analyses in clinical settings. Thirdly, some patients were undergoing treatment, which introduced further confounding effects.

## 4. Conclusions

In conclusion, this clinical investigation underscores the potential of breath analysis as a non-invasive method for the early detection of lung cancer. The study meticulously enrolled a diverse cohort comprising 14 lung cancer patients, 14 non-lung cancer controls with various medical conditions, and 3 tuberculosis patients, adhering to matching criteria including age, gender, smoking history, and comorbidities. The study employed a systematic breath sampling protocol with a BIO-VOC sampler, coupled with advanced VOC analysis using Thermal Desorption–Gas Chromatography–Mass Spectrometry (TD-GC/MS). The study’s findings indicate that a composite biomarker composed of eight specific volatile organic compounds (VOCs)—hexanal, heptanal, octanal, benzaldehyde, undecane, phenylacetaldehyde, decanal, and benzoic acid—demonstrated reliable detection of lung cancer in both the initial discovery set and subsequent validation set. Additionally, the results suggest that analyzing breath VOCs may assist in distinguishing between cases of tuberculosis and lung cancer, presenting a potential clinical benefit. In the discovery set, these eight VOC biomarkers achieved an AUC of 0.85, with sensitivity and specificity values of 82% and 76% respectively, underlining its potential as a diagnostic tool. These findings were further validated in an independent cohort, where the biomarker demonstrated an AUC of 0.78, with sensitivity and specificity values of 78% and 64% respectively. Moreover, the study delves into the underlying biochemical pathways contributing to elevated aldehyde levels in the breath of lung cancer patients, attributing this phenomenon to overactivated Alcohol Dehydrogenase (ADH) pathways in cancerous tissues. While acknowledging certain limitations, including factors such as sample size, population demographics, and potential confounding variables, the study’s outcomes strongly advocate for the potential of breath analysis in lung cancer diagnosis. Additionally, the study suggests its utility in differentiating tuberculosis from lung cancer.

These findings hold promise for advancing the development of non-invasive diagnostic tools for both lung cancer and tuberculosis [36]. Further research endeavors and rigorous validation are warranted to fully realize the clinical potential of breath analysis in the realm of lung cancer diagnosis.

## Figures and Tables

**Table 1:** Clinical characteristics of the discovery set.

	Lung Cancer	Controls	TB
Total Number	14	14	3
Sex (M/F)	10/4	10/4	2/1
Mean Age ± SD	67 ± 10	67 ± 7	51 ± 20
Smoking History			
Current smokers	4	4	2
Ex-smokers	8	8	0
Pack-years of smoking	38.7	38.6	40
Non-smokers	2	2	1
Histology			
Adenocarcinoma	9		
Squamous cell carcinoma	3		
Unknown	2		
Stage			
1 and 2	3		
3 and 4	10		
Unknown	1		

**Table 2:** Clinical characteristics of the validation set.

	Lung Cancer	Controls
Total Number	18	16
Sex (M/F)	14/4	13/3
Mean Age ± SD	66 ± 9	64 ± 12
Smoking History		
Current smokers	3	3
Ex-smokers	10	9
Pack-years of smoking	37.5	30
Non-smokers	5	4
Histology		
Adenocarcinoma	16	
Squamous cell carcinoma	1	
Unknown	1	
Stage		
1 and 2	2	
3 and 4	15	
Unknown	1	

**Table 3:** Fold change (lung cancer/control) and *p* value of 8 compounds in the discovery set and validation set.

	**Discovery Set**		**Validation Set**	
	**Fold Change**	***p* Value**	**Fold Change**	***p* Value**
Hexanal	1.8	0.00000001	1.3	0.05
Heptanal	2.0	0.000005	1.2	0.27
Octanal	2.1	0.000006	1.3	0.26
Decanal	2.4	0.000003	1.8	0.17
Benzaldehyde	1.7	0.000009	1.3	0.38
Phenylacetaldehyde	1.4	0.001	1.3	0.03
Undecane	2.0	0.00002	1.4	0.08
Benzoic Acid	3.5	0.00003	1.4	0.21

## Data Availability

Data are contained within the article.
